# Renal Rehabilitation: Present and Future Perspectives

**DOI:** 10.3390/jcm13020552

**Published:** 2024-01-18

**Authors:** Masahiro Kohzuki

**Affiliations:** 1 President and Chairman, Department of Health Sciences, Yamagata Prefectural University of Health Sciences, Yamagata 990-2212, Japan; kohzuki@med.tohoku.ac.jp; Tel./Fax: +81-23-686-6601; 2 Professor Emeritus, Department of Health Sciences, Tohoku University Graduate School of Medicine, Sendai 980-8575, Japan; 3 Chairman of the Board of Directors, International Society of Renal Rehabilitation, Sendai 980-8575, Japan; 4 Former Chairman of the Board of Directors, Japanese Society of Renal Rehabilitation; Tokyo 150-0043, Japan

**Keywords:** chronic kidney disease, exercise, rehabilitation, renal protection, cardio-renal syndrome, Japanese Society of Renal Rehabilitation, International Society of Renal Rehabilitation

## Abstract

Chronic kidney disease (CKD) is a global health problem. In patients with CKD, exercise endurance is decreased, especially as renal dysfunction advances. This is due to the combined effects of protein-energy wasting, uremic acidosis, and inflammatory cachexia, which lead to sarcopenia and are aggravated by a sedentary lifestyle, resulting in a progressive downward spiral of deconditioning. Renal rehabilitation (RR) is a coordinated, multifaceted intervention designed to optimize a patient’s physical, psychological, and social functioning, as well as to stabilize, slow, or even reverse the progression of renal deterioration, improving exercise tolerance and preventing the onset and worsening of heart failure, thereby reducing morbidity and mortality. This review focused on the history and benefits of RR in patients with CKD. Based on current evidence, RR is an effective, feasible, and safe secondary prevention strategy in CKD. RR is a promising model for a new field of rehabilitation. Therefore, efforts to increase RR implementation rates are urgently needed.

## 1. Introduction

Chronic kidney disease (CKD) is a global health problem. For example, the number of CKD patients in Japan is more than 11% of the total population. The number of patients undergoing hemodialysis (HD) in Japan is 349,700, corresponding to 1 in 359 of the total population in 2021 [1].

Further, CKD is associated with premature aging. Patients with CKD are characterized by frailty, osteoporosis, muscle wasting, cardiovascular hypertrophy, and vascular calcification [2]. Patients with CKD with dialysis have a very high mortality risk due to cardiovascular diseases such as chronic heart failure, and sedentary patients with CKD undergoing dialysis have an even higher mortality risk [3]. An independent, graded association has been found between a reduced glomerular filtration rate (GFR) and the risk of cardiovascular events, hospitalization, and death [4]. In addition to being a strong cardiovascular risk factor, physical inactivity is associated with an increased risk of rapid decline in renal function in patients with CKD [5].

In patients with CKD, exercise endurance is decreased, and this becomes more distinct as renal dysfunction advances. This is due to the combined effects of protein-energy wasting (PEW), uremic acidosis, and inflammatory cachexia, which lead to sarcopenia and are aggravated by a sedentary lifestyle. Collectively, these factors result in a progressive downward spiral of deconditioning [2].

Renal rehabilitation (RR) is a coordinated, multifaceted intervention designed to optimize a patient’s physical, psychological, and social functioning, as well as to stabilize, slow, or even reverse the progression of renal deterioration, improving exercise tolerance and preventing the onset and worsening of heart failure, thereby reducing morbidity and mortality. [6,7]. This review focused on the history and benefits of RR in patients with CKD.

## 2. CKD and Physical Inactivity

Physical inactivity is a major health problem. Regular exercise is important for maintaining good health and preventing chronic diseases. Moreover, an association between physical inactivity and poor outcomes in patients with CKD has been well established [7,8,9]. Patients with CKD typically engage in lower levels of physical activity than the general population, which can induce a catabolic state, including reduced neuromuscular functioning, exercise tolerance, and cardiorespiratory fitness.

**Figure 1:**
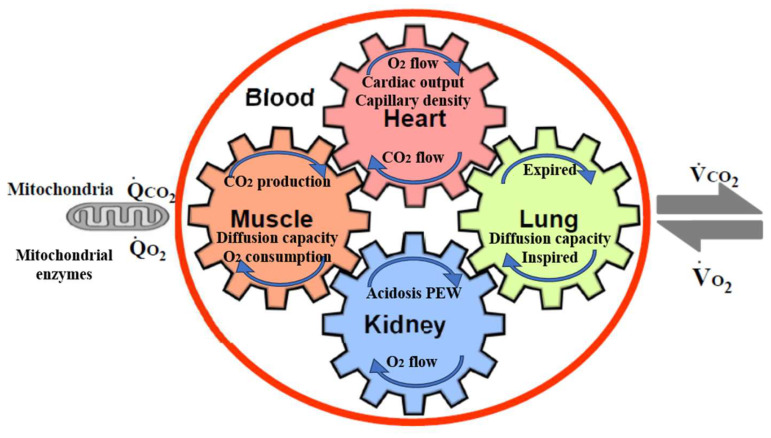
The five major determinants of VO_2_ max, peak VO_2_, and their relationships in CKD (based on [14]). The gears represent the functional interdependence of the physiological components of the system. Cardiac output, pulmonary diffusion capacity, oxygen-carrying capacity, renal function, metabolic acidosis, and other peripheral limitations, such as muscle diffusion capacity, mitochondrial enzymes, and capillary density, are all examples of VO_2_ max determinants. VO_2_, O_2_ uptake; VCO_2_, CO_2_ output; QCO_2_, CO_2_ production; QO_2_, O_2_ consumption by cells.

In addition to physical inactivity, cardiorespiratory (CR) fitness is an important consideration, as a strong predictor of mortality [10,11]. Low CR fitness has a particularly high risk of death compared to that for other common risk factors, such as dyslipidemia, hypertension, and diabetes. [12]. CR fitness is defined as the ability of the respiratory and circulatory systems to supply oxygen during physical activity, and is usually expressed as the maximal oxygen uptake (VO_2_ max) or peak oxygen uptake (peak VO_2_) during exercise testing [13]. VO_2_ max is the maximum rate of oxygen consumption attainable during physical exertion. A similar measure is peak VO_2_, which is the measurable value from a session of physical exercise. Be it incremental or otherwise, it could match or underestimate the actual VO_2_ max. Figure 1 shows the five major determinants of VO_2_ max, peak VO_2_, and their relationships in CKD [14]. The gears in Figure 1 represent the functional interdependence of the physiological components of the system. Pulmonary diffusion capacity, cardiac output, oxygen-carrying capacity, renal function, and other peripheral limitations such as capillary density, muscle diffusion capacity, and mitochondrial enzymes are all examples of VO_2_ determinants.

An increase in O_2_ utilization by the muscles (QO_2_) is achieved by an increase in ventilation, an increase in pulmonary blood flow by recruitment and vasodilatation of pulmonary blood vessels, an increase in cardiac output (stroke volume and heart rate), dilatation of selected peripheral vessels, and increased extraction of O_2_ from the blood perfusing the muscles. O_2_ is taken up from the alveoli (VO_2_) in proportion to the pulmonary blood flow and degree of O_2_ desaturation of hemoglobin in the pulmonary capillary blood. Metabolic acidosis in CKD patients promotes protein-energy wasting (PEW) [15], muscle protein wasting, and reducing protein synthesis [16,17]. In addition to sarcopenia by physical inactivity, PEW, metabolic acidosis, angiotensin II accumulation, and myostatin overexpression in uremia also contribute to the pathogenesis of muscle wasting, especially in CKD [18]. Erythropoietin can increase VO_2_ max in humans [19].

## 3. Chronic Effects of Exercise in CKD Animal Models

Evidence of the benefits of regular exercise in long-term conditions is accumulating. Further, the influence of chronic exercise on renal function must be considered, as acute exercise causes proteinuria, reduction in renal blood flow, and reduction in GFR. As it is shown clinically, sudden severe exercise decreases renal function [20,21]. However, such intense exercise cannot be performed for long. In other words, it is important to look at the effects of exercise over the long term. However, there is insufficient information regarding the influence of chronic exercise on renal function and the effect of exercise in pre-dialysis patients with CKD. For instance, the optimal duration and intensity of exercise for CKD patients with pre-dialysis has not yet been determined.

Since the late 1990s, my colleagues and I have published several papers in this field. We assessed the renal effects of moderate treadmill chronic exercise in several CKD rat models and found that exercise does not worsen renal function and had renoprotective effects in some rat models, such as a remnant kidney model of genetic hypertensive rats [22], 5/6-nephrectomized rats [23], diabetic nephropathy rats [24], and Zucker diabetic rats [25].

## 4. Chronic Effect of Exercise in Patients with CKD Undergoing Dialysis

In the Dialysis Outcomes and Practice Patterns Study, patients with CKD undergoing dialysis who were regular exercisers had higher health-related quality of life (HR-QOL), sleep quality scores, and physical functioning; the study also reported fewer limitations in physical activities than those who were not regular exercisers [8]. Regular exercise was also correlated with more positive patient effects and fewer depressive symptoms [8]. Further, in models extensively adjusted for demographics and comorbidities, the mortality risk was lower with regular exercise and at facilities with more regular exercisers [8].

Meta-analyses of randomized controlled trials (RCTs) have reported that regular exercise training in patients with CKD undergoing dialysis (HD) has benefits in physical function, aerobic capacity, dialysis adequacy, depressive symptoms, and HR-QOL [26,27,28,29,30]. Additionally, a meta-analysis of combined aerobic and resistance exercises (CARE) performed during HD by Liu et al. [31] found that CARE improved the peak oxygen uptake; performance on the six-minute walking test; 60-s and 30-s sit-to-stand tests; dialysis adequacy; scores on five (out of eight) domains and the physical component summary for HR-QOL on the Medical Outcomes Study Short Form-36; blood pressure; and hemoglobin levels in patients on maintenance HD compared to those with usual care. Further, subgroup analysis showed that intradialytic CARE resulted in the amelioration of more evaluated outcomes than non-intradialytic CARE, with the exception of handgrip strength and hemoglobin levels [31]. The authors concluded that CARE is an effective way to improve physical function, aerobic capacity, HR-QOL, and dialysis adequacy in patients on maintenance HD [31].

## 5. Chronic Effect of Exercise in Pre-Dialysis Patients with CKD

In the first RCT on the effect of exercise in CKD patients with pre-dialysis, reported by Baria et al. [32], sedentary pre-dialysis men with CKD (creatine-based estimated GFR) were randomly assigned to home-based exercise group, a center-based exercise group, or control group. In the exercise group, aerobic exercise was done three times per week for 12 weeks. During the study period, eGFRcreat was increased by 3.6 ± 4.6 mL/min (*p* = 0.03) in the center-based group, but remained unchanged in the control group [32]. Further, in a single-blind randomized controlled study of the effects of moderate-intensity regular exercise on renal function and indices of cardiovascular risk in patients with stages 3–4 CKD by Greenwood et al., there was a significant difference in the rate of change in eGFRcreat between exercise and usual care groups, with the exercise group showing a slower decline in function [33].

Chen et al. reported the associations between walking, mortality, and renal replacement therapies (RRTs), such as peritoneal dialysis, HD, and kidney transplantation, in patients with stages 3–5 CKD [34]. Among 6363 patients (mean age, 70 years), 1341 (21.1%) reported walking as their most common form of exercise. The rate of mortality was lower in walking patients than in non-walking patients (2.7 vs. 5.4 per 100 person-years). Similarly, the incidence of RRT was significantly lower in walking patients than in non-walking patients (22 vs. 32.9 per 100 person-years) [34]. Further, walking was associated with lower mortality and RRT risk on multivariate regression. The adjusted sub-distribution hazard ratio (SHR) of walking was 0.67 (*p* < 0.001) for overall mortality and 0.79 (*p* < 0.001) for the risk of RRT. Further, the SHRs of overall mortality were 0.83, 0.72, 0.42, and 0.41. Thus, walking is the most popular form of exercise for patients with CKD and is associated with a lower risk of mortality and RRT. [34].

**Figure 2:**
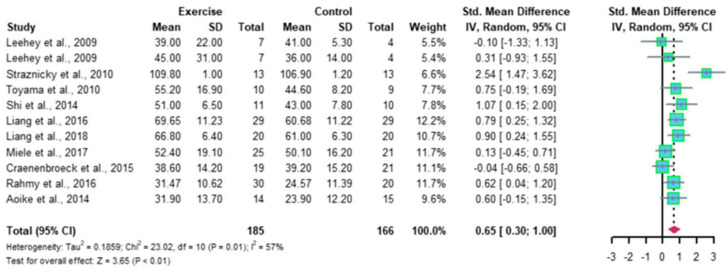
Meta-analysis of the effect of aerobic exercise on eGFR (adapted from [35]). The results indicate that regular aerobic exercise significantly improves the eGFR. eGFR, estimated glomerular filtration rate; CI, confidence interval; SD, standard deviation.

**Figure 3:**
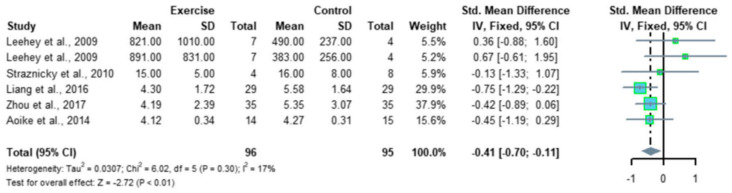
Meta-analysis of the effect of aerobic exercise on 24-h urinary protein excretion (adapted from [35]). The results indicate that regular aerobic exercise significantly improves the 24-h urine protein volume in patients with chronic kidney disease. CI, confidence interval; SD, standard deviation.

Recently, Ma et al. reported a meta-analysis of 12 RCTs, comprising 410 patients with CKD; the results indicated that regular aerobic exercise significantly improves the estimated GFR (eGFR), and the levels of serum creatinine, daily urinary protein excretion, and serum urea nitrogen in CKD patients. Further, a single exercise session of more than 30 min was associated with significantly improved eGFR (*p* < 0.01), including walking and running, but not cycling, as exercise modalities were associated with significantly improved serum creatinine levels (*p* < 0.05) in CKD patients (Figure 2 and Figure 3) [35]. Thus, regular aerobic exercise has beneficial effects on the eGFR (especially with durations longer than 30 min), serum creatinine (especially with walking or running as the modality), daily urinary protein excretion, and blood urea nitrogen levels in CKD patients [35].

## 6. Chronic Effect of Exercise in Pre-Dialysis CKD Patients with Acute Myocardial Infarction (AMI)

CKD is common in patients with diabetes mellitus, occurring in approximately 40% of cases. Diabetes mellitus is also an important risk factor for cardiovascular diseases; however, CKD is an important mediator of this risk [36]. Kidney function is closely linked to heart function. Renal dysfunction/disease may initiate, accentuate, or precipitate cardiac dysfunction/disease, and vice versa [37].

**Figure 4:**
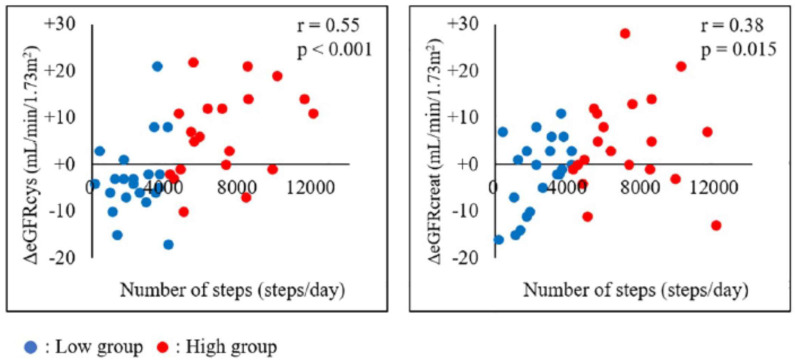
Association between the number of steps and ΔeGFRcys or ΔeGFRcreat (adapted from [38]). The association between the number of steps and eGFRcys or eGFRcreat in all patients is shown [38]. Pearson’s correlation analysis revealed significant correlations between the number of steps and both eGFR parameters, with a higher correlation between ΔeGFRcys and the number of steps than between ΔeGFRcreat and the number of steps. eGFRcreat, creatine-based estimated glomerular filtration rate; eGFRcys, cystatin C-based estimated glomerular filtration rate.

Combined, renal dysfunction worsens the prognosis after AMI. My colleagues and I investigated the association between physical activity levels and renal function changes in AMI patients [38]. Renal function was measured using the cystatin C-based eGFR (eGFRcys), which is independent of muscle mass. Patients were stratified into a low exercise group (2335 ± 1219 steps/day) and a high exercise group (7102 ± 2365 steps/day). eGFRcys was significantly increased after 3 months of exercise in the high exercise group, whereas no significant change was observed in the low exercise group. Further, the change in eGFRcys was greater in the high exercise group (+6.7 mL/min/1.73 m^2^) than in the low exercise group (−2.9 mL/min/1.73 m^2^) [38]. The physical activity level was positively associated with renal function changes, demonstrating that high physical activity levels may suppress renal function decline in AMI patients. Figure 4 indicates the association between the number of steps and eGFRcys or eGFRcreat [38]. Pearson’s correlation analysis revealed significant correlations between the number of daily steps and both eGFR parameters. Furthermore, the coefficient was greater for the correlation between ΔeGFRcys and the number of daily steps (r = 0.55, *p* < 0.001) than between ΔeGFRcreat and the number of daily steps (r = 0.38, *p* = 0.015). As previously indicated, changes in serum creatinine levels can be caused by changes in skeletal muscles through exercise, highlighting the importance of using eGFRcys as an indicator of renal function [39,40]. A recent prospective study verified the association between physical activity levels and renal function in patients with CKD [39]. The results were similar to those in the above-mentioned study and indicated that maintaining a high level of physical activity in daily life leads to the suppression of renal function deterioration [41]. However, our study was the first to show an association between physical activity level and changes in renal function after the onset of AMI using an accelerometer and eGFRcys. Our findings support the importance of interventions to maintain a high physical activity level as a strategy for renal protection in patients with AMI. Future research should verify the long-term effects of physical activity on renal function in patients with AMI.

There is immense potential for research in the field of cardio-nephrology, in terms of diagnosis, prognosis, complication risk evaluation, and the utilization of novel therapeutic approaches for CKD patients and associated cardiovascular complications. However, significant advancements have been made to improve patient care and outcomes in patients with CKD [42].

## 7. Mechanisms of Renal Protection by Chronic Exercise

Increasing evidence indicates that chronic exercise has beneficial effects on chronic inflammation, muscle and bone strength, CR fitness, and metabolic markers in patients with CKD or kidney transplants [43]. However, the mechanisms underlying these benefits have received little attention, and the available clinical evidence is mainly from small, short-duration (<12 weeks) exercise studies [43]. Bishop et al. summarized that the available data, suggest exercise results in shifts towards a less inflammatory immune cell profile, reduced monocyte infiltration into the adipose tissue, and enhanced activity of the NRF2 pathway, may underlie improvements in inflammatory biomarkers [43]. Exercise-mediated increases in nitric oxide release and bioavailability, left ventricular remodeling, myocardial fibrosis, and reductions in angiotensin II accumulation in the heart may contribute to improvements in left ventricular hypertrophy. Although exercise stimulates an anabolic response in skeletal muscles in CKD, satellite cell activation and increases in mitochondrial mass seem to be impaired in this population. Exercise-mediated activation of the canonical “Wnt” pathway may lead to bone formation and improvements in the levels of the bone-derived hormones, Klotho, and fibroblast growth factor 23 (FGF23) [43].

The musculoskeletal and cardiopulmonary systems are the predominant organ systems typically considered in exercise studies. The importance of renal physiology in physical activity and exercise can be easily overlooked. However, over the past 30 years, research has revealed the relevance of renal function in regulating physiological responses to exercise, providing insights on how exercise can alter the pathophysiology of kidney diseases [44]. In support of this, the editors of the American Journal of Physiology-Renal Physiology have encouraged submissions to the Call for Papers on “Exercise and the Kidney in Health and Disease” [44].

## 8. Barriers to Exercise Participation among Patients with CKD

Unfortunately, the role of physical activity in CKD has been largely overlooked, and the provision of rehabilitation and exercise for CKD patients lags behind that for cardiac and pulmonary services. The Kidney Disease Outcomes Quality Initiative (K/DOQI) clinical practice guidelines comment that all dialysis patients should be counseled and regularly encouraged by nephrology and dialysis staff to increase their physical activity [45].

Delgado et al. reported the survey on exercise counseling to nephrologists [46]. On multivariate analysis, older nephrologists (odds ratio 3.3) and more physically active nephrologists (odds ratio: 5.5) were more likely to counsel CKD patients about exercise [46]. Responses related to less counseling behavior included a lack of confidence in the ability to discuss physical activity.

Delgado and colleagues also reported that patients with CKD undergoing dialysis were interested in exercise [47]. However, 92% of participants reported at least one barrier to physical activity. The most commonly reported barriers were shortness of breath and fatigue on non-dialysis and dialysis days. On multivariate analysis, a greater number of barriers was associated with a lower level of physical activity. Additionally, a lack of motivation was associated with a lower physical activity level. Endorsement of too many medical problems and insufficient time on dialysis days were also associated with lower physical activity levels in an adjusted analysis [47].

The location of exercise is also an important factor that influences adherence. In CKD patients with dialysis, exercise during dialysis programs have been found to achieve higher adherence rates than home exercise or supervised programs on non-dialysis days [48].

## 9. Present Status of RR

### 9.1. Societies and Meetings

Several international working groups have been established to address the physical inactivity contributes to the burden of disease in CKD patients [49,50,51,52]. The Japanese Society of Renal Rehabilitation (JSRR) was established in 2011 to promote and disseminate RR in Japan. I was the first president of the board of directors from 2011–2020. The number of members in the JSRR has increased annually; as of 1 April 2023, the number of individual members was 3005, and the number of facilities or supporting members was 170. Annual scientific meetings of the JSRR are held, and approximately 300 papers are presented every year. The number of participants in seminars for RR professionals is expected increase to over 8000 in 2023.

In the JSRR, we consider RR to include five major components: exercise training, diet and fluid management, medication and medical surveillance, education, and psychological and vocational counseling [6,7]. The JSRR uses a comprehensive approach to RR, including physical exercise and psychological, vocational, and dietary counselling. The JSRR has a newsletter, an official English journal (Renal Replacement Therapy), and an official Japanese journal (The Japanese Journal of Renal Rehabilitation).

In March 2018, in order to improve the quality of RR and educate RR professionals in Japan, the JSRR established a certification program for a Registered Instructor of RR (RIRR). The minimum requirements for candidates of the RIRR certification examination are as follows: (1) possess a certification or degree for any of the following positions: physician, nurse, physical therapist, occupational therapist, medical engineer, clinical psychologist, clinical laboratory technician, and exercise trainer; (2) be a member of the JSRR for more than 2 years; (3) have a minimum of 1 year of experience in a RR program or equivalent; and (4) submit 10 case reports on rehabilitation for patients with CKD. To date, 738 members have undergone this examination. Those who qualify for RIRR can provide exercise therapy and RR to dialysis patients and claim medical fees for exercise therapy during dialysis. Therefore, obtaining the RIRR would give the instructor an advantage in finding employment at dialysis facilities and rehabilitation facilities.

In November 2019, the Global Renal Exercise Network (GREX) held a meeting in Canada which was attended by international clinicians and researchers, including myself. Taking a global perspective, the meeting highlighted Japanese, Canadian, and other regional examples of policies developed regarding exercise and rehabilitation [53]. In the meeting, it was noticed that Japan leads the field of RR in four ways: Japan has a national society (JSRR), certification for RR (RIRR), guidelines for RR, and a National Health Insurance Reimbursement program for RR.

I was invited to give a lecture on “Rehabilitation for Visceral Impairment (Renal rehabilitation)” at the ISPRM2020 meeting in Orlando, Florida; viewing this lecture was mandatory for a specialist renewal from the Association of Academic Physiatrist Rehabilitation from 1 July 2020 to 1 July 2023.

Finally, as there was no international society for RR, the International Society of Renal Rehabilitation (ISRR) was established in 2020 to promote and disseminate RR. An annual scientific meeting of the ISRR is held every March.

### 9.2. Guidelines of RR

The American College of Sports Medicine and KDIGO have published guidelines for exercise testing and prescription, as well as for specific methods and cautions regarding exercise therapy for patients with CKD [54,55].

I first edited and published a book titled “Renal Rehabilitation” [6]. In order to clarify the programs and effectiveness of RR, the Renal Rehabilitation Guideline Preparation Committee was launched in 2016 under JSRR, which created a guideline in accordance with the “Minds Handbook for Clinical Practice Guideline Development 2014” [54,55]. Six recommendations were created for various kidney disorders, with groups addressing CKD, nephritis, nephrosis, dialysis, and kidney transplantation [56,57]. All the recommendation grades were determined through a consensus conference. The exercise prescriptions for individuals with kidney disease which are recommended by JSRR are shown [55,56]. In brief, the exercise prescription consists of three exercises: an aerobic exercise, a resistance exercise, and a flexibility exercise. The frequency (F), intensity (I), time (T), and type (T) of each exercise is as follows.

In aerobic training, F (3–5 d·wk^−1^), I (moderate intensity (40–59% VO_2_R (oxygen uptake reserve), RPE 12–13 on a scale of 6–20), T (20–60 min of continuous activity; however, if this cannot be tolerated, use 3–5 min bouts of intermittent exercise, aiming to accumulate 20–60 min·d^−1^), T (prolonged, rhythmic activities using large muscle groups (e.g., walking, cycling, swimming)) [55,56].

In the resistance exercise, F (2–3 d·wk^−1^), I (65–75% 1-RM (one repetition maximum). The performance of 1-RM is not recommended; estimate 1-RM from a ≧3-RM test), T (a minimum of one set of 10–15 repetitions, with a goal in most patients to achieve multiple sets. Choose 8–10 different exercises targeting the major muscle groups) and T (machines, free weights, or bands) [55,56].

In the flexibility exercise, F (2–3 d·wk^−1^), I (static: stretch to the point of tightness or slight discomfort; PNF (proprioceptive neuromuscular facilitation): 20–75% of the maximum voluntary contraction), T (60 s per joint for static (10–30 s hold per stretch); 3–6 s contraction followed by 10–30 s assisted stretch for PNF), and T (static or PNF) [55,56].

### 9.3. National Health Insurance Reimbursement

Infrastructure and reimbursement systems for cardiac rehabilitation exist in many countries. In contrast, heath care systems in most countries have no or limited coverage of for exercise training for CKD patients. Therefore, CKD patients are often unable to overcome barriers to exercise and are unable to find appropriate exercise facilities [58].

To our knowledge, Japan is the only country in the world with a national health insurance system for RR [59,60]. In April 2016, Japan’s Ministry of Health, Labor and Welfare decided to expand the scope of rehabilitation to diabetic patients with CKD stages 4 to 5 through the world’s first national health insurance reimbursement system. Each hospital or clinic can receive one additional medical fee per month for each CKD patients with RR. Eligible conditions include: 50% or more of patients suppressing the increase in serum creatinine or serum cystatin, a 20% or more decrease in daily urine protein excretion, or a 30% reduction in the slope of 1/creatinine or 1/cystatin. The content of the exercise should be in accordance with JSRR guidelines [55,56], and it is desirable to have RIRR qualifications. Nature Review in Nephrology introduced Japan’s national health insurance system for JSRR and RR [59].

Additionally, from April 2018, exercise training for diabetic patients with CKD stage 3b is covered by the National Health Insurance. Furthermore, from April 2022, exercise training for CKD patients with HD is covered by the National Health Insurance. Under this system, if a patient receives exercise therapy instructions for 20 min or more during dialysis, a medical fee is allowed for up to 90 days from the start of the fee. The conditions are that the instructor has an RIRR (valid forever), or if the instructor is without an RIRR, they must attend a JSRR seminar and pass the examination (valid for 3 years). A qualified physician, physical therapist, or an occupational therapist can supervise 15 inpatients and 20 outpatients at a time, and a qualified nurse can supervise 5 inpatients and 8 outpatients at a time.

## 10. Future perspectives of RR: Adding Life to Years and Years to Life

Evidence regarding the effectiveness of components other than exercise therapy in renal rehabilitation, such as education, is still emerging. However, in order to encourage inactive CKD patients to exercise, education about the effects of exercise is a prerequisite. In addition, the importance of dietary therapy that increases the effectiveness of exercise therapy, the importance of dietary therapy that does not cause sarcopenia or frailty, and how to continue exercise are very important when performing RR. JSRR’s annual scientific meeting features many presentations on these topics. In the future, it is expected that more effective educational methods, more effective dietary therapy menus, and more effective exercise therapy menus will be established regarding RR. The next RR guideline by JSRR will include a comprehensive list of factors beyond exercise therapy.

**Figure 5:**
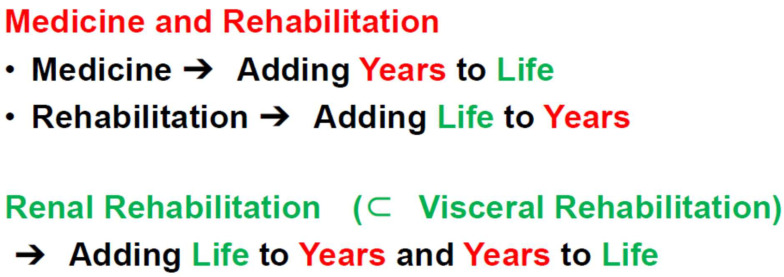
Renal rehabilitation is a new target in rehabilitation (based on [61]). Medical science basically aims to add years to life by increasing life expectancy. Rehabilitation generally aims to add life to years by helping patients with impairment to achieve and use their full physical, mental, and social potential. However, accumulating evidence suggests that rehabilitation in patients with visceral impairments, such as renal, cardiac, and pulmonary impairments, can not only improve exercise performance and HR-QOL, but can also increase survival (adding life to years and years to life).

As a super-aged society has emerged, the number of individuals with multiple morbidities and disabilities who require rehabilitation has increased more rapidly than expected [60]. Medical science basically aims to add years to life by increasing life expectancy. Rehabilitation generally aims to add life to years by helping patients with impairment achieve and use their full physical, mental, and social potential. However, accumulating evidence suggests that rehabilitation for patients with visceral impairments, such as renal, cardiac, and pulmonary impairments, can not only improve exercise performance and HR-QOL, but can also increase survival (Figure 5) [61]. Therefore, modern comprehensive rehabilitation for patients with CKD does not simply aim to add life to years, but to add life to years and years to life, which is a new rehabilitation concept [61].

## 11. Conclusions

RR is a coordinated, multifaceted intervention designed to optimize a patient’s physical, psychological, and social functioning in addition to stabilizing, slowing, or even reversing the progression of renal deterioration, improving exercise tolerance and preventing the onset and worsening of heart failure, thereby reducing morbidity and mortality. RR is an effective, feasible, and safe secondary prevention strategy in CKD and is a promising model for a new field of rehabilitation.

Future large RCTs should focus more on the effects of rehabilitation and exercise programs, as these topics and exercise types have not been studied as extensively as cardiovascular and pulmonary rehabilitation. Moreover, efforts to increase RR implementation rates are urgently needed.

## Data Availability

Not applicable.
